# Role of *Achyranthes aspera* in neurodegenerative diseases: current evidence and future directions

**DOI:** 10.3389/fphar.2025.1511011

**Published:** 2025-04-09

**Authors:** Huaiqing Luo, Siwen Wei, Shujun Fu, Li Han

**Affiliations:** ^1^ Key Laboratory of Model Animals and Stem Cell Biology in Hunan Province, Hunan Normal University Health Science Center, Changsha, Hunan, China; ^2^ Institute of Interdisciplinary Studies, Hunan Normal University Health Science Center, Changsha, Hunan, China; ^3^ Department of Immunology, Jishou University School of Medicine, Jishou, Hunan, China; ^4^ Hunan Provincial Key Laboratory of New Pharmaceutical Preparation, Changsha Medical University, Changsha, Hunan, China

**Keywords:** *Achyranthes aspera*, neurodegenerative diseases, Alzheimer’s disease, Parkinson’s disease, Huntington’s disease, Amyotrophic lateral sclerosis, neuroprotection

## Abstract

Neurodegenerative diseases are caused by the progressive degeneration of neurons and/or their myelin sheaths, ultimately leading to cognitive and motor dysfunction. Due to their complex pathogenesis and the limited efficacy of therapeutic drugs, these diseases have attracted significant attention. *Achyranthes aspera*, belongs to family Amaranthaceae, has been extensively used in the traditional and folk medicines for the treatment of various ailments. Modern research has revealed that *Achyranthes aspera* possesses various pharmacological effects, including cardiocerebrovascular protection, immune regulation, antioxidation, and anti-aging. Furthermore, the neuroprotective effects of *Achyranthes aspera* have been confirmed by numerous scientific studies. This review focuses on the primary pharmacological effects and mechanisms of *Achyranthes aspera* in the prevention and treatment of neurodegenerative diseases, as well as their potential application prospects. This review aims to provide insights into the potential clinical applications and research directions of *Achyranthes aspera* in neurodegenerative diseases.

## 1 Introduction

Neurodegenerative diseases are a group of diseases characterized by progressive neuronal loss and neurological dysfunction (Wilson et al., 2023). These diseases primarily include Alzheimer’s disease (AD), Parkinson’s disease (PD), Huntington’s disease (HD), and Amyotrophic lateral sclerosis (ALS). Aging is a major risk factor for the onset of neurodegenerative diseases (Hou et al., 2019). With the intensification of population aging, the incidence of neurodegenerative diseases is rapidly increasing., and their high mortality and disability rates severely impact patients’ physical and mental health, placing a significant burden on both families and society. Although modern medicine has made some progress in the treatment of neurodegenerative diseases, current therapeutic approaches mainly focus on symptom relief and slowing disease progression. There is still a lack of effective curative treatments, and the drugs available on the market often have notable toxic side effects (Erkkinen et al., 2018). Therefore, there is an urgent need to elucidate the pathogenesis of these diseases and to seek safer and more effective treatment strategies. Given the complexity of the mechanisms underlying neurodegenerative diseases, an increasing body of research suggests that traditional Chinese medicine, with its multi-target and multi-pathway approach to disease prevention and treatment, holds significant advantages in the management of neurodegenerative diseases.

*Achyranthes aspera*, also known as *Achyranthes aspera* Linnacus, belongs to the Amaranthaceae family and the Achyranthes genus. It includes the wild species of Achyranthes bidentata, Achyranthes longifolia and *Achyranthes aspera*. *Achyranthes aspera* is a traditional herbal medicine that has been used in China for thousands of years and mainly belongs to perennial plants (HU et al., 2021). *Achyranthes aspera* is different from Cyathula officinalis Kuan, radix Achyranthis bidentatae, and Chinese eupatorium root, which are different in origin, active ingredients, and efficacy (He, 2013). *Achyranthes aspera* was initially documented in the “Compendium of Materia Medica.” It possesses a taste that is sweet, slightly bitter, and slightly acidic. It is characterized as having a cool temperament and an affinity for the liver and kidney meridians. *Achyranthes aspera* has therapeutic benefits that include promoting blood circulation to remove stasis, clearing heat and detoxicating, and inducing diuresis for treating stranguria. It is primarily used for treating conditions such as cold and fever, tonsillitis, diphtheria, mumps, urinary stones and nephritis edema (Yang, 2001). Modern pharmacological studies have revealed that *Achyranthes aspera* exhibits a variety of pharmacological activities, including antioxidant, anti-aging, anti-inflammatory, and immunomodulatory effects. Its active components, such as saponins, flavonoids, and polypeptides, have shown significant roles in neuroprotection and neural repair (Gawande et al., 2017; Viswanatha et al., 2019), demonstrating promising therapeutic potential for neurodegenerative diseases. This article reviews the mechanisms of action of *Achyranthes aspera* extracts and its active components in neurodegenerative diseases, aiming to provide new perspectives and theoretical foundations for the development and utilization of *Achyranthes aspera* and related research on the treatment of neurodegenerative diseases.

## 2 Overview of the pathogenesis of neurodegenerative diseases

**FIGURE 1:**
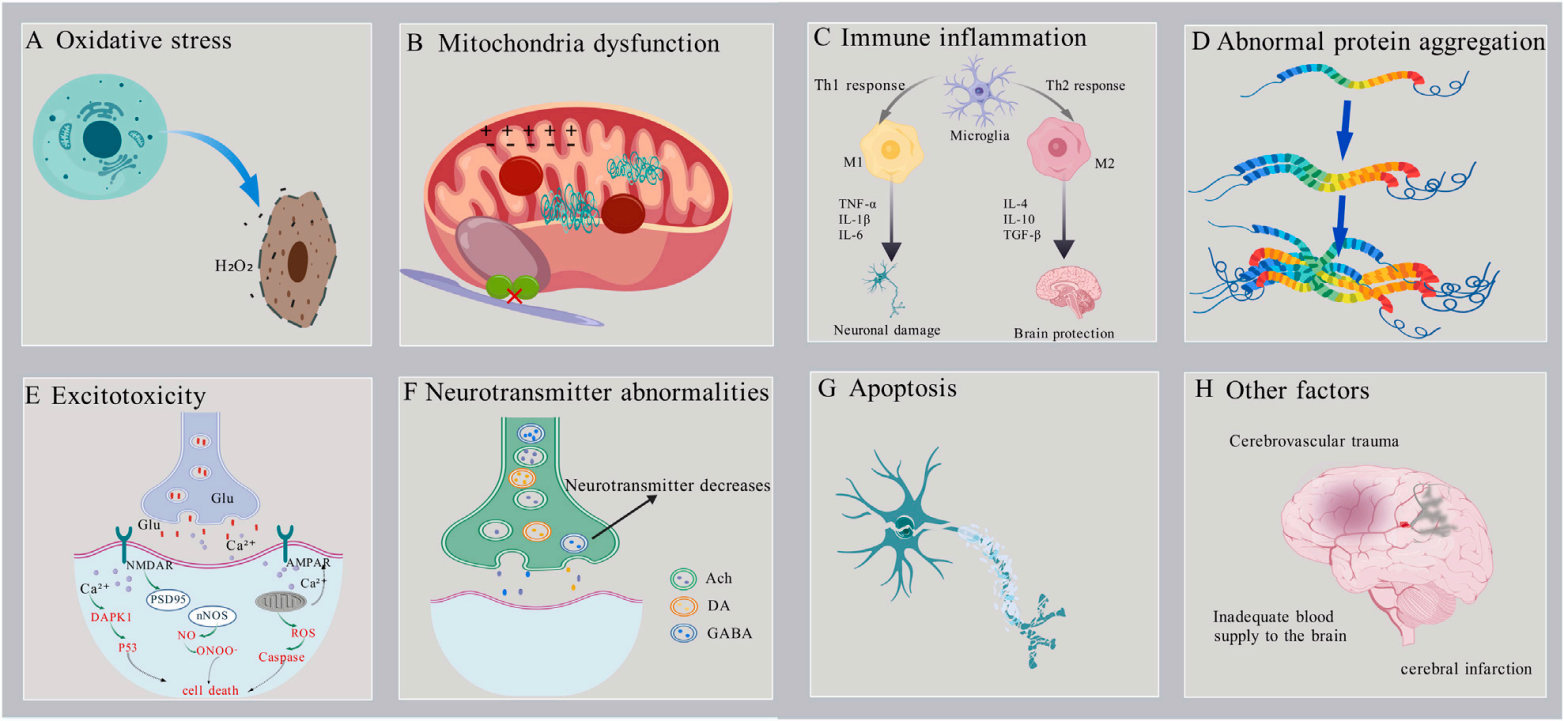
Schematic diagram of the pathogenesis of neurodegenerative diseases.

Neurodegenerative diseases have a significant impact on the daily life and health of patients, prompting increased research into their pathogenesis. Previous studies have identified several factors that contribute to the development of neurodegenerative diseases, including oxidative stress, mitochondrial dysfunction, excitotoxins, immune inflammation, Ca^2+^ imbalance and apoptosis, (Figure 1).

### 2.1 Oxidative stress

Oxidative stress is the result of an imbalance between oxidation and antioxidant activity. This imbalance leads to the overproduction of free radicals or the failure to remove them efficiently, resulting in the accumulation of reactive oxygen species (ROS) in the body and subsequent causing cell damage (Figure 1A). Research indicates that oxidative stress is closely associated with the pathogenesis of various neurodegenerative diseases, including Alzheimer’s disease (AD), Parkinson’s disease (PD), Huntington’s disease (HD), and amyotrophic lateral sclerosis (ALS) (Lin and Beal, 2006; Scarian et al., 2024). In neurodegenerative diseases, excessive production of ROS leads to lipid peroxidation, protein oxidation, and DNA damage, which can trigger neuronal dysfunction and death. For instance, in the brains of patients with AD, there are observed abnormalities in mitochondrial DNA (mtDNA) and impaired oxidative phosphorylation (Bazzani et al., 2022). In addition, oxidative stress exacerbates neuronal damage by activating inflammatory responses and apoptotic pathways (Merelli et al., 2021; Singh et al., 2019). Therefore, reducing ROS levels represents a critical therapeutic strategy for the treatment of neurodegenerative diseases.

The antioxidant enzyme system and antioxidants are two crucial protective mechanisms by which the brain combats oxidative stress. The antioxidant enzyme system primarily consists of superoxide dismutase (SOD), catalase (CAT), and glutathione peroxidase (GSH-Px). SOD catalyzes the dismutation of superoxide radicals into hydrogen peroxide, while catalase and GSH-Px effectively eliminate the generated hydrogen peroxide, preventing cellular damage caused by excessive H2O2 concentrations (Salim, 2017). Antioxidants, including glutathione, vitamin C, and uric acid, can directly or indirectly scavenge oxygen free radicals, thereby reducing neuronal damage during oxidative processes (Feitosa et al., 2018). Additionally, antioxidants can chelate redox-active metals such as iron, copper, and zinc, thereby reducing the interaction of Aβ proteins and Tau proteins with these metals in the brains of AD patients. This reduces the aggregation of Aβ protein and the hyperphosphorylation of Tau, mitigating the damage caused by toxic proteins to the neuronal cells (Singh et al., 2019). Therefore, the neurological damage caused by oxidative stress can be alleviated by increasing the activity of low molecular antioxidants and the level of antioxidant enzymes such as SOD and GSH-Px.

### 2.2 Mitochondrial dysfunction

Mitochondria are essential for energy production and metabolism in the human body. They have a critical function in cell survival, necrosis, apoptosis, and maintaining calcium ion homeostasis. Additionally, mitochondria are the primary source of ROS. Age-related abnormalities in mitochondrial function and structure can lead to neuronal damage and apoptosis. Consequently, mitochondrial dysfunction is also a significant pathogenic mechanism underlying neurodegenerative diseases (Savu and Moisoi, 2022) (Figure 1B). Mitochondrial dysfunction occurs in the early stages of numerous neurodegenerative diseases. It not only disrupts the process of oxidative phosphorylation, leading to abnormal energy production and metabolism, but also increases the generation of ROS, inducing oxidative stress damage. Additionally, it causes imbalances in calcium ion concentrations, triggering a series of neuronal damage responses. Studies have found that AD patients exhibit defects in mtDNA and abnormalities in oxidative phosphorylation, along with an increased number of mitochondria and structural abnormalities, such as the presence of lamellar bodies and crystalline inclusions (Bazzani et al., 2022) In PD patients, dopaminergic neurons show significant mitochondrial dysfunction. Mitochondrial dysfunction is closely related to the pathological processes of PD, including the death of dopaminergic neurons, the aggregation of α-synuclein, and oxidative stress (Zong et al., 2024). Therefore, protecting mitochondrial function by improving mitochondrial structure, regulating the balance of mitochondrial fission, fusion, and autophagy may provide a new therapeutic approach and strategy for neurodegenerative diseases.

### 2.3 Immune inflammation

Existing evidence suggests that the overactivation of immune cells, the persistence of inflammatory responses, and the imbalance of immune reactions can all contribute to neuronal damage and death (Luchicchi et al., 2021; Zhang et al., 2024) and death (Figure 1C). Hence, modulating the activity of the immune system could serve as a novel approach to treating neurodegenerative diseases. Studies have shown that in mice with neurodegenerative diseases such as ALS and AD, concurrent immune deficiency accelerates disease progression, whereas restoring immune system function slows the course of the disease. (Beers et al., 2008).

Neurodegenerative diseases can be triggered by abnormalities in bothinnate and adaptive immune (DeMaio et al., 2022; Rajesh and Kanneganti, 2022). Innate immunity, which mainly consists of microglia and astrocytes, is responsible for monitoring and maintaining neuronal health. They ensure normal brain function by clearing cellular debris, regulating neuronal function, and modulating inflammatory responses. However, when microglia are repeatedly activated by inflammation inflammatory stimuli (e.g., aggregated forms of Aβ), they shift from the anti-inflammatory M2 phenotype to the pro-inflammatory M1 phenotype (Samar et al., 2023). This transition leads to the release of large amounts of inflammatory factors, such as interleukin-6 (IL-6), TNF-α, and IL-1, which accelerate the production and accumulation of neurotoxic proteins. These neurotoxic proteins, in turn, further activate glial cells, resulting in the release of more inflammatory factors, then causing neuronal damage and death. Furthermore, this worsens the pathological progression of neurodegenerative disorders like AD, PD, and HD (Rauf et al., 2022). Monocytes may be involved in the pathogenesis of PD, as there is an evidence of dysregulation of peripheral blood monocytes in PD patients. Specifically, an elevated number of pro-inflammatory monocytes is observed alongside the activation of the CCR2-CCL2 axis in PD (Tansey et al., 2022).

T cells and B cells are the primary mediators of adaptive immunity, enabling targeted immune responses against specific antigens. Early research has shown that T cells in the central nervous system (CNS) are often considered harmful, especially in neuroinflammatory diseases. In these diseases, abnormal T cells (with an increased Th1/Th2 ratio) contribute to the expansion of neuroinflammation by interacting with neuroglial cells in the brain and releasing pro-inflammatory mediators (Absinta et al., 2021; Yoshimura et al., 2023). Blocking the entry of T cells into the brain or inhibiting their activation can significantly reduce neurological degeneration in mice with Alzheimer’s disease (Dai and Shen, 2021). T cells not only directly contribute to neurodegeneration in neurodegenerative diseases but also influence the progression of the disease by modulating the immune response. Certain subsets of T cells,such as Treg and Th2 cells, inhibit the excessive activation of other immune cells, thereby limiting inflammation and reducing damage to neurons (Ellwardt et al., 2016). Therefore, adjusting the equilibrium of T lymphocytes can have a positive impact on the central nervous system. Individuals with CNS inflammation exhibit a significant increase in the number of B lymphocytes in the cerebrospinal fluid (Moccia et al., 2022), while patients with PD show decreased levels of B lymphocyte subsets in their peripheral blood (Ahn et al., 2021). Hence, it is crucial to suppress neuroinflammation, particularly the aberrant activation of microglia and astrocytes, and regulate neuroimmunity as effective strategies to hinder and postpone the onset and progression of neurodegenerative disorders.

### 2.4 Abnormal protein aggregation

The aggregation of abnormal proteins can form toxic oligomers and fibrils, which interfere with normal cellular functions and ultimately lead to neuronal death, contributing to the development of various neurodegenerative diseases (Figure 1D) (Olzscha et al., 2011). Examples include the aggregation of Aβ and tau proteins in AD (Thal et al., 2015), the abnormal aggregation of α-synuclein in PD (Mehra et al., 2019) and the aggregation of Huntingtin (Htt) protein in HD (Jimenez-Sanchez et al., 2017). In addition, abnormal protein aggregation exacerbates neuronal damage by inducing oxidative stress and inflammatory responses (Liu et al., 2023; Poewe et al., 2017; van Roon-Mom et al., 2008). Therefore, inhibiting the aggregation of abnormal proteins and promoting their degradation may be an important strategy for the treatment of neurodegenerative diseases.

### 2.5 Excitotoxicity

As the most important excitatory neurotransmitter in the brain, glutamate can participate in physiological activities such as excitatory synaptic transmission and regulation of neurotransmitter release after binding to the receptors. However, high extracellular glutamate concentration will over-activate the N-methyl-D-aspartic acid (NMDA) receptors and α-amino-3-hydroxy-5-methyl-4-isoxazole propionic acid (AMPA) receptor. This leads to neuronal membrane depolarization, causing a massive influx of Ca^2+^, Na^+^, Cl^−^, and water, causing cellular osmotic swelling, lysis, and ultimately neuronal aging, damage, and death (Figure 1E) (Nussbaum et al., 2012). Excitotoxicity is associated with neurodegenerative diseases such as AD’,PD,HD,and ALS (Bruijn et al., 2004; Pan et al., 2020; Singh et al., 2024). For example, in AD patients, overstimulation of excitatory transmitters (e.g., glutamate) causes an overinflux of Ca^2+^ in neurons. This triggers mitochondrial calcium overload, leading to mitochondrial dysfunction, insufficient energy supply to neurons, and the release of large amounts of reactive oxygen species (ROS). This induces oxidative stress damage and calcium dysregulation, ultimately triggering neuronal apoptosis (Zott et al., 2019). Therefore, promoting glutamate uptake or inhibiting glutamate release and its receptor activation may mitigate excitotoxic neuronal damage caused by excessive glutamate concentrations.

### 2.6 Neurotransmitter abnormalities

The normal processes of neurotransmitter release and reuptake are crucial for the conduction of neural signals and the stability of neural networks. However, with aging, abnormalities in the synthesis, release, metabolism, and reuptake of neurotransmitters may occur, which are closely associated with the development and progression of various neurodegenerative diseases (Figure 1F). Studies have shown that acetylcholine (Ach) levels are significantly lower in AD patients. In PD patients, the degeneration of dopaminergic neurons in the substantia nigra results in a marked decrease in dopamine (DA) levels in the striatum (Griffith et al., 2008; Salama et al., 2024). γ-Aminobutyric acid (GABA) is the primary inhibitory neurotransmitter and plays a crucial role in regulating neuronal excitability. In HD patients, the degeneration of GABAergic neurons in the striatum leads to reduced GABA levels. This results in neuronal hyperexcitability, triggering motor disorders and cognitive decline (Kocak et al., 2022). These abnormalities in neurotransmitter levels can lead to neuronal dysfunction and death. In addition, abnormal neurotransmitter levels exacerbate neuronal damage by inducing oxidative stress and inflammatory responses (Burbulla et al., 2017; Maczurek et al., 2008). Therefore, modulation of neurotransmitter levels may represent an important therapeutic strategy for neurodegenerative diseases.

### 2.7 Apoptosis

Apoptosis is a genetically controlled programmed cell death process that is essential for normal development and maintenance of homeostasis in organisms. However, excessive induction of apoptosis can also lead to neuronal death, so apoptosis is an important pathogenesis of neurodegenerative diseases (Figure 1G) (Gupta et al., 2021; Yuan et al., 2019). Autophagy and apoptosis are two primary mechanisms for degrading misfolded proteins, playing a critical role in maintaining cellular quality control by eliminating abnormal proteins. Under stress conditions, the ability of autophagy to degrade misfolded proteins is impaired, leading to the accumulation of abnormal proteins, induction of apoptosis, and ultimately neuronal death. Upregulating autophagy through autophagy inducers can reduce the accumulation of misfolded proteins and thereby slow the progression of neurodegenerative diseases (Zhang et al., 2021). Key proteins regulating apoptosis include B-cell lymphoma-2(Bcl-2), Caspase-3, and Bcl-2-associated X protein (Bax). Enhancing the expression of the anti-apoptotic protein Bcl-2 and inhibiting the expression of Caspase-3 and the pro-apoptotic protein Bax through synthetic drugs or natural small molecules can suppress neuronal apoptosis and provide protective effects against apoptosis-induced neuronal damage (Li et al., 2020; Xu et al., 2024).

In addition to the above mechanisms, abnormal function of cerebrovascular endothelial cells, cerebrovascular injury, cerebral insufficiency, cerebral infarction, cerebral ischemia-reperfusion injury (Figure 1H) (Ren et al., 2023); decreased expression of the neuronal nuclear transcription factor c-fos, protein kinase PKB, and the heat shock protein HSP70, and reduced tolerance and self-protection of neuronal cells against cerebral ischemia can cause neuronal cell injury and the development of neurodegenerative diseases (Bendszus et al., 2023; Poll et al., 2020; Yu et al., 2021; Zhao and Wang, 2020).

## 3 The active ingredient and mechanism of *Achyranthes aspera* protection against neurodegenerative diseases

### 3.1 Chemical composition of *Achyranthes aspera*

Up to now, more than 70 compounds have been isolated from *Achyranthes aspera* (He et al., 2017; Krishnaveni and Thaakur, 2006). These components primarily include triterpenoids and their saponins, steroids, organic acids, alkaloids, and flavonoids. The chemical constituents of *Achyranthes aspera* are mainly isolated from its roots and rhizomes, leaves, seeds, young shoots, and aerial parts. Gas chromatographic analysis showed that the leaves of *Achyranthes aspera* also contained relatively rich volatile oil (Bi et al., 2023; Krishnaveni and Thaakur, 2006).

### 3.2 Effective components and related mechanisms of *Achyranthes aspera* in the prevention and treatment of neurodegenerative diseases

#### 3.2.1 Triterpenoid saponins

**FIGURE 2:**
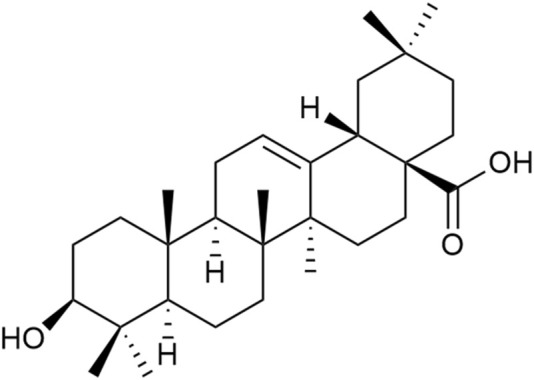
Structure of the mother nucleus of triterpenoid saponin in *Achyranthes aspera*.

Triterpenoid saponins have anti-inflammatory, antioxidant, and neuroprotective effects (Chen et al., 2022). Currently, the triterpenoid saponins isolated from *Achyranthes aspera* are mainly saponins derivatives with oleanolic acid (OA) as the aglycone. A total of 39 compounds (1–39) have been identified, and their core structures are illustrated in Figure 2 (Luo et al., 2020).

Triterpenes and their saponins, particularly OA and its derivatives, have been widely studied in the field of neuroprotection. These compounds can treat neurological diseases by alleviating excitotoxicity, Ca^2+^ overload, mitochondrial dysfunction, and blood-brain barrier permeability, as well as regulating immunity, anti-inflammatory, antioxidant, anti-apoptosis, and promoting angiogenesis and neurogenesis (Chen et al., 2022). The studies reported that OA (100 mg/kg) protects dopamine neurons by attenuating striatal microglial activation in a PD rat model induced by 6-hydroxydopamine (6-OHDA) (Mabandla et al., 2015; Msibi and Mabandla, 2019). The pretreatement with OA (20 μM) can exhibited antioxidative, anti-inflammatory and anti-apoptosis activities via decreasing GSH, raising the activity of SOD and catalase, reducing the release of IL-6 and TNF-α in PC12 cells (Tsai and Yin, 2008).

A significant pathological feature of AD is Aβ plaque deposition. OA (10 mg/kg) has been shown to inhibit Aβ25-35-induced cellular and synaptic toxicity (Wang et al., 2018), Aβ levels, and neuronal apoptosis, and reduce ROS levels by regulating uncoupling protein-2 (UCP2) expression through stanniocalcin-1 (STC-1), which can alleviate oxidative stress damage and play a role in the treatment of AD (Guo et al., 2020). Astrocytes and microglia are the primary immune cells in AD. In Aβ-activated astrocytes, OA (40 μM) pretreatment inhibits the transcription and release of inflammatory cytokines IL-6, TNF-α, and IL-1β, as well as inhibits neuroinflammation and neurotoxicity triggered by Ca^2+^ overload in co-cultured neurons (Zhang et al., 2018). OA (1 μM, 5 μM, and 10 μM) also inhibits inflammatory responses triggered by microglia overactivation (Castellano et al., 2019). In addition, OA (10 mg/kg) can promote the proliferation and differentiation of neural stem cells to enhance hippocampal neurogenesis, improve cell survival, thereby achieving neuroprotection and repair. These help ameliorate Aβ-induced cognitive and memory impairment (Lin et al., 2021). The oleanolic acid derivative, oleanolic acid saponin, increases acetylcholine levels and improves cognitive function by inhibiting the activity of acetylcholinesterase (AChE) (Puri et al., 2024).

#### 3.2.2 Ketosteroid

**FIGURE 3:**
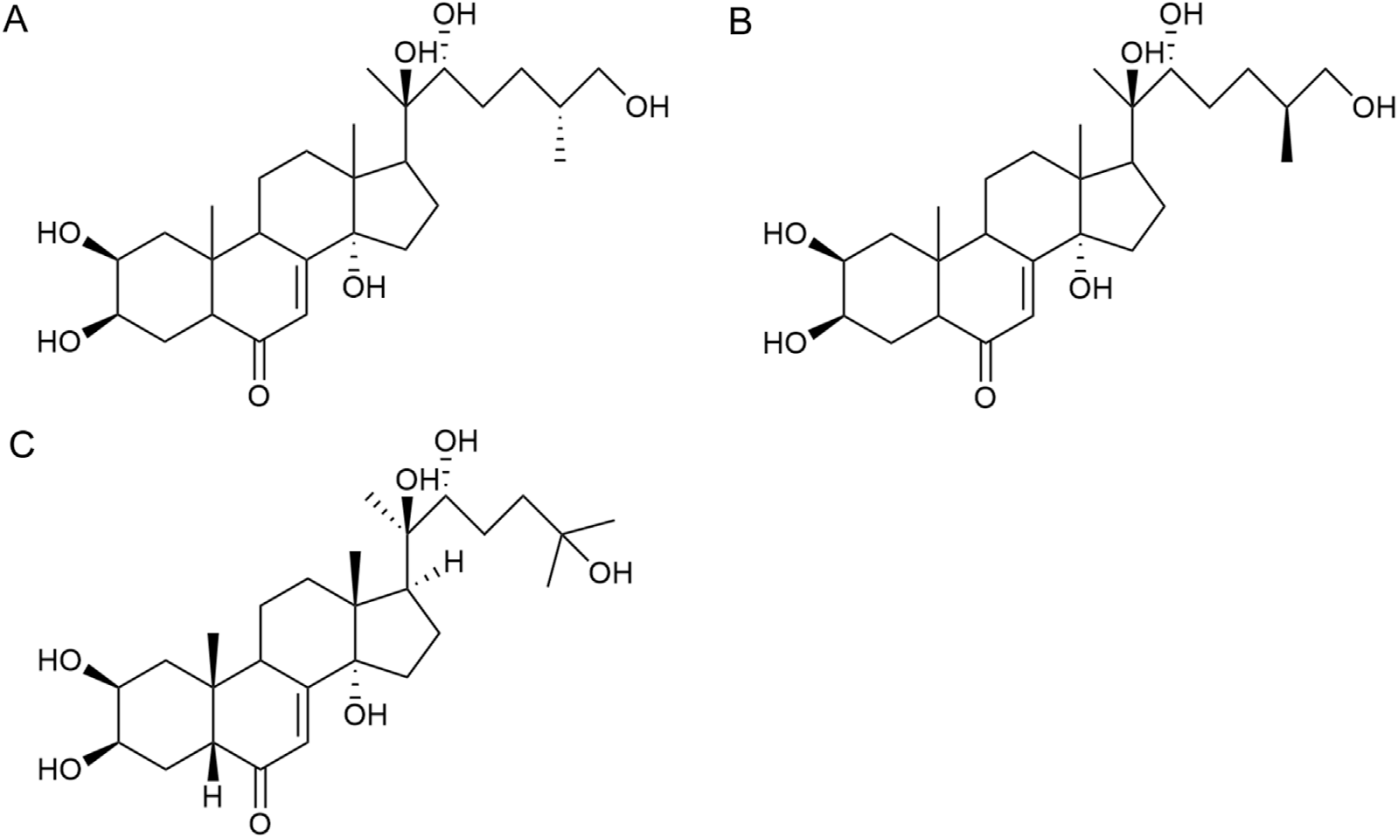
Steroid compounds in *Achyranthes aspera*. **(A)** 25R-achyranosterone. **(B)** 25S- achyranosteron. **(C)** β--ecdysterone.

Ketosteroid compounds are also the main active ingredients of *Achyranthes aspera*. Currently, there are seven ketosteroid compounds isolated from *Achyranthes aspera*, primarily including 25R-inokosterone (Figure 3A), 25S-inokosterone (Figure 3B), and β-ecdysterone (Figure 3C) (Meng, 2004).

Inokosterone, especially β-ecdysterone, have demonstrated potential and research value in the treatment of neurological disorders. Ecdysterone exhibits neuroprotective effects including antioxidant, anti-inflammatory, neurotransmitter modulation, inhibition of neuronal apoptosis, and promotion of neuronal regeneration and repair. Various factors, such as oxidative stress and neuronal loss, have been associated with neurodegenerative diseases like PD. β-Ecd (0.8 μM) has a protective effect on rotenone-induced neurotoxicity in PC12 cells, which can enhance the viability of PC12 cells through the Akt/Nrf2 pathway and reduce rotenone-induced apoptosis by decreasing Bax expression, caspase-9 activity, and caspase-3 activity (Liu et al., 2016).

β-Ecd also has a good therapeutic effect on AD. Ecd (5 mg/kg) administrated for 4 weeks in micehas been shown to scavenge oxygen free radicals as well as improve cognitive function, restore hippocampal SOD, CAT, and GRx activities, and inhibit neuronal loss in the cerebral cortex and hippocampus (Gholipour et al., 2022a; Gholipour et al., 2022b; Xing et al., 2024). BACE1 is an aspartic protease that cleaves amyloid precursor protein (APP) to generate neurotoxic Aβ (Mullard, 2017). β-Ecd strongly binds to the active site of BACE1, inducing a conformational change from an open to a closed form, thereby blocking substrate binding. Even 500 nM of the compound completely blocks the enzyme activity. Furthermore, β-Ecd strongly inhibits the formation and aggregation of Aβ fibrils and promotes the degradation of Aβ fibrils. (Chakraborty and Basu, 2017; Yang et al., 2010).

Neuronal hyperexcitability is a common phenomenon in neurodegenerative diseases. Ecd prevents glutamatergic excitotoxicity by enhancing mTOR/Akt/PI3K signaling activity to significantly reduce cortical cell death and exerts cortical neuron protectant for the prevention and treatment of dementia, mental and behavioral disorders (Wu et al., 2017). In the brain, dysfunction of the GABAergic system also disrupts t the balance between excitation and inhibition, resulting in neurodegeneration (Piot et al., 2023). Factors such as Aβ, BACE1, ApoE4, TREM2 mutations, and overactivated glial cells can all contribute to GABAergic system dysfunction (Bi et al., 2025; Cheng et al., 2023; Kaarniranta et al., 2023). Therefore, abnormalities in GABAergic system may serve as a common target for multiple aberrant signaling pathways in AD. Dysfunction of the GABAergic system, in turn, promotes the spread of Aβ and tau pathology, further exacerbating cognitive impairment in AD patients (Rossano et al., 2024). Ecd can act on the regulatory sites of GABAergic receptors to enhance GABAergic inhibition in cortical neurons, thereby reducing excitotoxicity and reducing neuronal cell death (Tsujiyama et al., 1995).

Ecd also possesses the ability to promote neuronal growth and repair. 200 mg/L ecd significantly increased the neurogenesis and differentiation of hippocampal neural stem cells, which is highly beneficial for stem cell transplantation in the treatment and injury repair of central nervous system disorders (Chu, 2008), β-ecd has been studied for its potential to improve symptoms of cerebral ischemia, alleviate cerebrovascular spasms, and enhance learning and memory capabilities, all of which hold significant importance for AD patients (Hindle et al., 2017).

#### 3.2.3 Flavonoids

**FIGURE 4:**
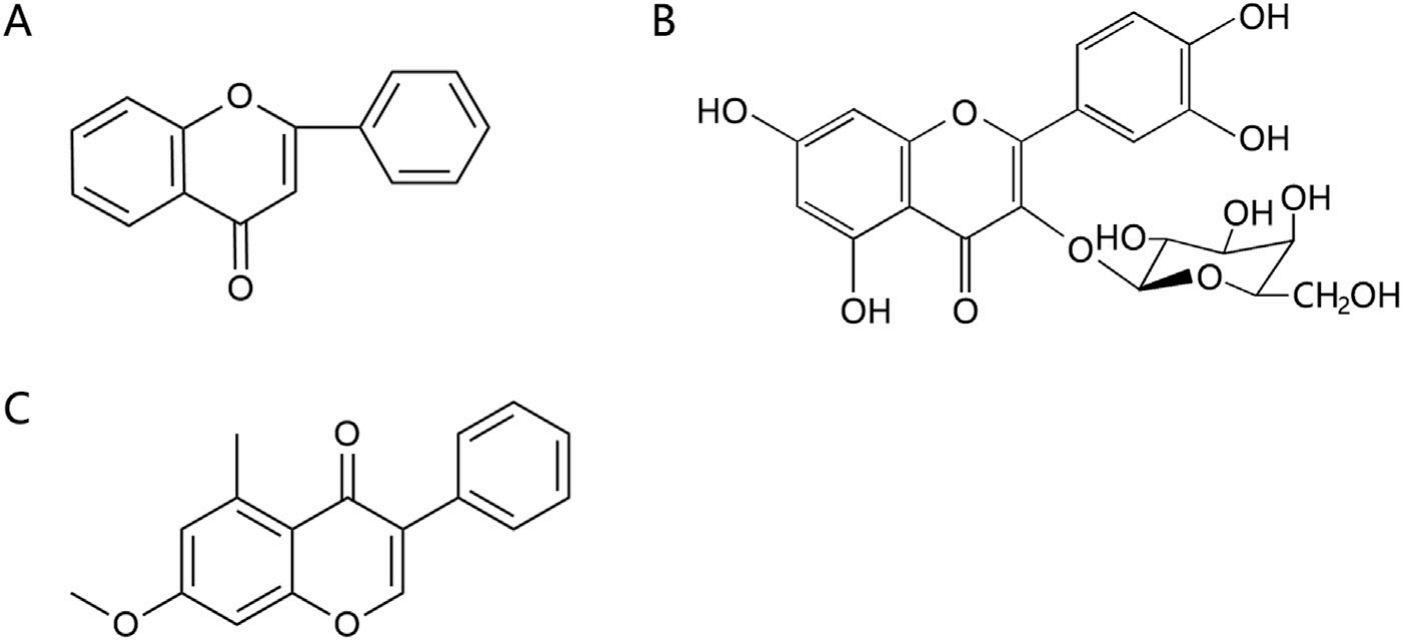
Flavonoids in *Achyranthes aspera*. **(A)** 2-phenylchromogen. **(B)** Hyperoside. **(C)** 5,2′-dimethoxy-6-(methoxymethyl)-7-hydroxy-isoflavonol.

**FIGURE 5:**
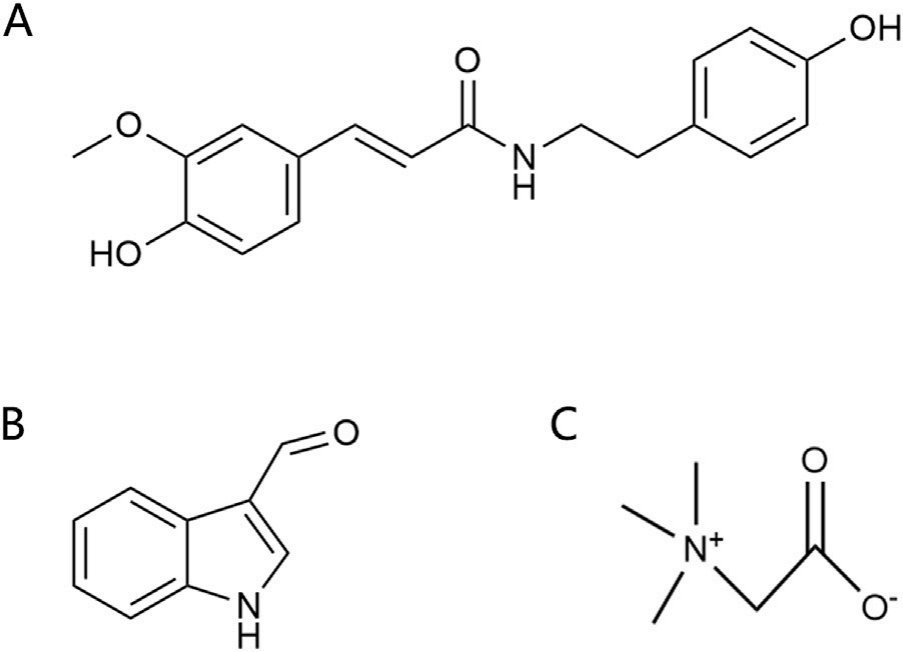
Alkaloids in *Achyranthes aspera*. **(A)** N-trans-feruloyl-tyramine. **(B)** Indole-3-carboxaldehyde. **(C)** Betaine.

Flavonoids are effective components of antioxidant and scavenging oxygen free radicals, as well as immunomodulatory, anti-inflammatory, anti-tumor, antibacterial, vascular-nourishing, and cardiovascular disease-preventing effects (Guan and Liu, 2016; Wang et al., 2023). Flavonoids are a class of yellow pigments derived from the flavone (2-phenylchromogen, as shown in Figure 4A) (Wen et al., 2010). The two main flavonoids found in *Achyranthes aspera* are hyperoside (HYP) (Figure 4B) and 5,2′-dimethoxy-6-(methoxymethyl)-7-hydroxy-isoflavonol (Figure 4C).

In the field of neuroprotection, HYP is the most extensively studied flavonoid, while other flavonoid components in *Achyranthes aspera* have not been reported in related studies. HYP, also known as quercetin-3-O-β-D-Nitrophenyl β-D-fucopyranoside, is alternatively referred to as hyperin or quercetin-3-galactoside. It is an important natural product belonging to the flavonol glycoside class, with a structure of quercetin-3-galactoside (Li, 2008). Clinically, HYP is used in many drugs, primarily for treating cardiovascular diseases. In addition, HYP exhibits a variety of effects, including anti-tumor, anti-aging, antidepressant, and anti-inflammatory properties. It also regulates the circulatory system, immune, digestive, and nervous system (Wang Q. et al., 2022).

In animal and cellular models of AD, HYP inhibits endoplasmic reticulum-related apoptotic pathways and attenuates Aβ-induced brain endothelial cell damage and blood-brain barrier (BBB) disruption, thus exerting a protective effect against AD (Liu et al., 2018; Liu et al., 2017). HYP (2.5, 5, 10, and 20 μM) also reversed Aβ-induced mitochondrial dysfunction, including elevation of mitochondrial membrane potential, reduction of reactive oxygen species (ROS) production and release of mitochondrial cytochrome c, and inhibition of caspase-9 and caspase-3, thereby inhibiting apoptosis (Zeng et al., 2011). Pretreatment with hyperoside for 30 min significantly increases cell viability, and morphological assessments of axonal damage supported that HYP pretreatment effectively reverses the toxic effects induced by Aβ and improves learning and memory deficits (Yi et al., 2022; Zeng et al., 2011). Aβ peptides can spontaneously aggregate into β-sheet-containing oligomers and fibrils, and the activation of this amyloid pathway alters the Ca^2+^ signaling, leading to neurotoxicity, which in turn leads to neuronal apoptosis (Angelova and Abramov, 2014). HYP (20, 40, 80 mg/kg) has been shown to exert neuroprotective effects in cellular or APP/PSEN1 double transgenic AD mouse models by anti-Aβ aggregation, BACE1 inhibition, reduction of Aβ plaques and GFAP levels in cortex and hippocampus. It also modulates Aβ-induced cell death by regulating Ca^2+^ signaling cascades and mitochondrial membrane potential, thereby providing neuroprotection (Song et al., 2023). Pharmacokinetic data confirmed that intranasal administration of HYP increased bioavailability in the mouse brain. Further *in vivo* studies demonstrated that it improved motor deficits, spatial memory, and learning ability in APP/PSEN1 mice (Song et al., 2023).

PD is characterized by the pathological loss of DA neurons in the nigrostriatal pathway, leading to insufficient DA release and resulting in both motor and nonmotor symptoms. HYP (100 μg/mL) reduced 1-Methyl-4-phenyl-1,2,3,6-tetrahydropyridine (MPTP)-mediated cytotoxicity in SH-SY5Y cells *in vitro*, while HYP (25 mg/kg) alleviated MPTP-induced motor symptoms *in vivo*, reduced levels of nitric oxide (NO), H_2_O_2_, and malondialdehyde (MDA), and mitochondrial damage in dopaminergic neurons (Xu et al., 2023). Additionally, HYP treatment elevated the levels of neurotrophic factors such as glial cell line-derived neurotrophic factor (GDNF), brain-derived neurotrophic factor (BDNF), and cerebral dopamine neurotrophic factor (CDNF) *in vivo* (Xu et al., 2023). HYP (2 μM) also inhibits oxidative stress-induced neuronal death by activating Nrf2-dependent heme oxygenase-1 (HO-1), significantly ameliorating 6-hydroxydopamine (6-OHDA)-induced neurotoxicity and providing protective effects against PD and related disorders (Kwon et al., 2019). HYP effectively inhibit microglia overactivation and inflammation. Studies have found that hyperoside significantly inhibits LPS-induced production of NO, IL-1β and TNF-α in BV2 microglia by suppressing the activation of p38 and NF-κB, as well as reducing iNOS expression, thereby exerting neuroprotective effects (Fan et al., 2017). Furthermore, HYP inhibits the NLRP3 inflammasome activation by upregulating pituitary adenylate cyclase-activating peptide (PACAP), thus effectively suppressing neuroinflammation to protect dopaminergic neurons and treat PD (Wang K. et al., 2022).

HYP was able to significantly inhibit corticosterone-induced Ca^2+^ overload in PC12 cells, suggesting its ability to protect neuronal cells by preventing intracellular Ca^2+^ overload (Zheng et al., 2012). Additionally, HYP enhances the body’s immunity by promoting the activity and function of immune cells. For example, it stimulates lymphocyte proliferation, enhances the phagocytic function of monocytes and macrophages, and increases the production of antibodies (Gao et al., 2010; Huang et al., 2009). These effects provide a theoretical basis for the role of HYP in other neurodegenerative diseases.

#### 3.2.4 Alkaloids

Alkaloids are another important active components in *Achyranthes aspera*, exhibiting a variety of pharmacological activities. Alkaloids compounds, primarily including N-trans-feruloyltyramine (NTF) (Figure 5A), indole-3-carboxaldehyde (I3A) (Figure 5B), and betaine (Figure 5C), exert neuroactive effects on the nervous system, such as sedation and anti-depression (Bhosale et al., 2011).

NTF has been demonstrated to be a potent antioxidant (Gao et al., 2019). In the human neuroblastoma cell line SK-N-SH, NTF (320 μM) pretreatment significantly reduced H_2_O_2_-induced ROS generation and attenuated H_2_O_2_-mediated cytotoxicity. It reversed H_2_O_2_-mediated increases in the expression of Bax and activated caspase-3 as well as the decrease in Bcl-2, suggesting that NTF ameliorates H_2_O_2_-induced intracellular ROS generation and reduces apoptosis. These protective effects of NTF may be beneficial for neurodegenerative diseases associated with oxidative stress (Gao et al., 2019; Tu et al., 2016). NTF also inhibits BACE1, Monoamine oxidase-B (MAO-B), tau hyperphosphorylation, and Aβ aggregation, thereby inhibiting neurodegenerative diseases, especially AD (Othman et al., 2022). The alkaloid I3A also inhibits Aβ and tau protein aggregation (Purgatorio et al., 2020).

Betaine exhibits a wide range of pharmacological effects and has shown therapeutic potential for cardiovascular disease, liver disease, kidney disease, and neuroprotection. It can be used in the prevention and treatment of neurodegenerative diseases such as AD and PD (Rosas-Rodríguez and Valenzuela-Soto, 2021). Betaine metabolism provides methyl for methionine synthesis, which regulates homocysteine (Hcy) levels, and blood Hcy is a biomarker of human health status, and high levels of Hcy are associated with a variety of diseases (Azzini et al., 2020; Hainsworth et al., 2016). Betaine can attenuate high Hcy-induced memory impairment, chronic stress, and oxidative stress by reducing matrix metalloproteinases (MMP) in the frontal cortex of the brain (Kunisawa et al., 2015; Xie et al., 2016) and reversing tau hyperphosphorylation, Aβ aggregation, and levels of the inflammatory factors IL-1β and TNFα (Sun et al., 2017). Betaine inhibited Aβ-induced neuroinflammation in microglia by inhibiting the activation of the NLRP3 inflammasome and NF-κB (Zhang and Jia, 2023). Betaine interacts with GABA receptors to activate the GABAergic system, thereby reducing memory impairment (Kunisawa et al., 2017). It also regulates inhibitory neurotransmission, such as glycine and glutamine (Knight et al., 2017). These mechanisms highlight the significant potential of alkaloids in the prevention and treatment of neurodegenerative diseases.

Additionally, polysaccharides in *Achyranthes aspera* exhibit immunomodulatory effects, which help enhance the body’s immune function and reduce inflammatory damage to the nervous system. *Achyranthes aspera* extract is beneficial to improve the immunity of dogs by stimulating B cells and inducing anti-inflammatory response (Lee et al., 2020). Achyranthes polysaccharides(ABPS)selectively enhance the Th1 immune response, control the proliferation of plasmodium, and prolong the survival of mice in subsequent plasmodium infection (Zhu et al., 2012). ABPS significantly enhanced humoral response and B lymphocyte proliferation and antagonized the immunosuppressive effects (Li, 2000). Studies have shown that ABPS can markedly enhance the immunomodulatory functions of macrophages (Wang et al., 2021), increase the activity of natural killer cells (NK cells) in immunocompromised mice, and elevate the expression of CD40, CD80, and CD86 on the cell surface, stimulate the activation of T lymphocytes, and thus enhance the humoral immunity and non-specific immunity of the body. Therefore, *Achyranthes aspera* can improve neurodegenerative diseases by stimulating the immune system and increasing the body’s immune function.

## 4 Conclusion

**FIGURE 6:**
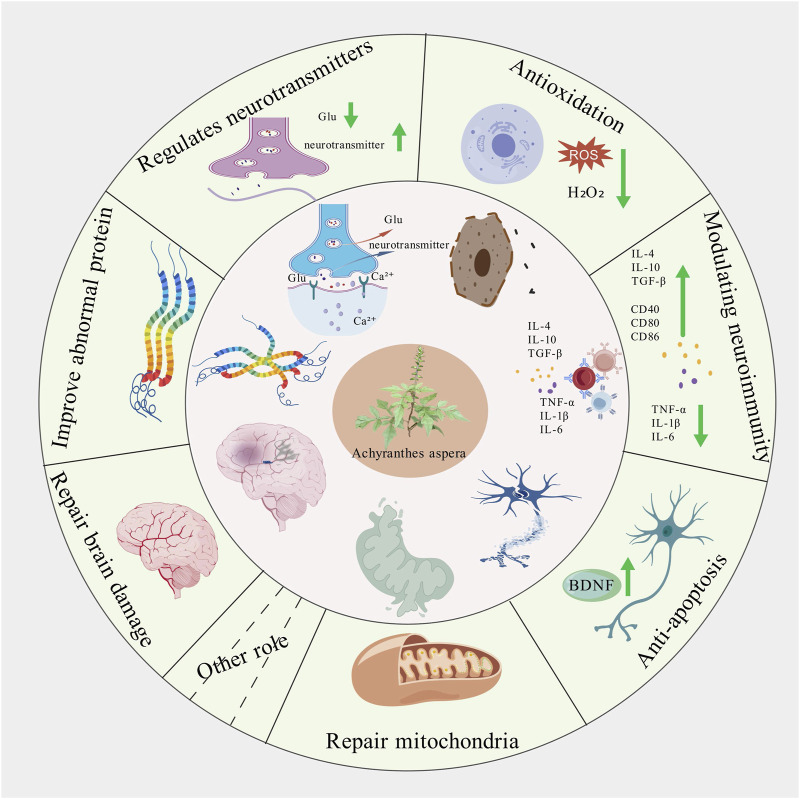
Schematic diagram of the effect of *achyranthes aspera* on neurodegenerative diseases.

*Achyranthes aspera* has been documented in the traditional medicine systems of multiple countries and regions, with a wide range of applications, which provids valuable clues for its further development and utilization. This review primarily summarizes the pharmacological mechanisms of the active components of *Achyranthes aspera* in neurodegenerative diseases. It has been found that *Achyranthes aspera* can reduce the production of free radicals in the brain tissue of patients with neurodegenerative diseases, upregulate the activity of antioxidant enzymes, and activate antioxidant stress pathways to exert antioxidative effects (Table 1). It inhibits the production of inflammatory factors by glial cells and suppresses inflammatory pathways to exert anti-inflammatory effects. It improves mitochondrial function by enhancing mitochondrial membrane potential, reducing the production of reactive oxygen species (ROS), and inhibiting the release of mitochondrial cytochrome c. It inhibits the expression of pro-apoptotic proteins to prevent cell apoptosis, reduces the production of neurotoxic proteins such as Aβ, α-synuclein (α-Syn), and phosphorylated tau proteins, prevents Ca^2+^ overload, ameliorates neurotransmitter dysregulation, and restores synaptic plasticity, thereby exerting neuroprotective effects. These findings demonstrate that *Achyranthes aspera* can exert therapeutic effects at multiple pathological stages of neurodegenerative diseases (Figure 6). Additionally, *Achyranthes aspera* has not shown significant acute toxicity or side effects in current usage, making it a promising candidate for the development of drugs targeting central nervous system disorders.

**TABLE 1:** Neuroprotective Components and mechanism in Achyranthes sinensis.

Categories	Main Ingredients	Chemical Structure (Molecular Formula)	Role	References
Triterpenoid saponins	Oleanolic Acid,OA	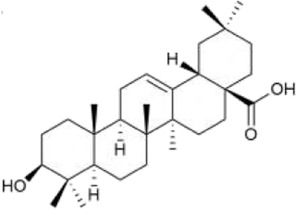	Attenuation of excitotoxicity Restoration of mitochondrial function Antioxidant and anti-inflammatory effects (IL-6, TNF-α, IL-1β suppression) Inhibition of apoptotic pathways Promotion of angiogenesis Suppression of pathological protein aggregation Regulation of neurotransmitter levels (e.g., AChE modulation) Increased dopamine levels	Guo et al. (2020), Mabandla et al. (2015), Puri et al. (2024), Tsai and Yin (2008)
Ketosteroid	25R-inokosterone	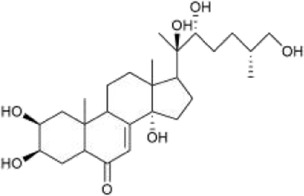	Antioxidant and anti-inflammatory activities	Meng (2004)
	25S-inokosterone	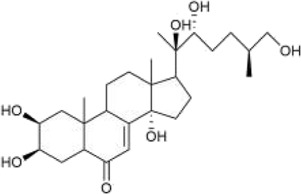	Antioxidant and anti-inflammatory activities	Meng (2004)
	β-ecdysterone	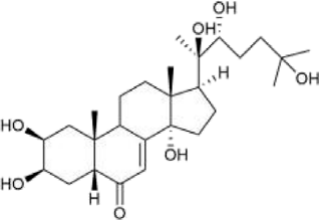	Antioxidant effects (*via* Akt/Nrf2 pathway activation) and anti-inflammatory properties Attenuation of glutamate-mediated excitotoxicity Inhibition of pathological protein aggregation (notably Aβ deposition) Regulation of neurotransmitter systems (particularly GABAergic modulation) Suppression of apoptotic pathways (via caspase-3 inhibition)	Chakraborty and Basu (2017), Liu et al. (2016), Wu et al. (2017), Yang et al. (2010)
Flavonoids	hyperoside	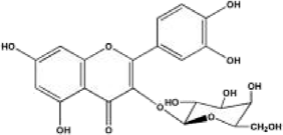	Inhibition of pathological protein aggregation (notably Tau phosphorylation and amyloid-beta [Aβ] deposition) Antioxidant effects and anti-inflammatory activity (through IL-1β and TNF-α suppression) Immunomodulatory effects Suppression of apoptotic pathways Restoration of mitochondrial function	Guan and Liu (2016), Liu et al. (2018), Wang et al. (2023), Xu et al. (2023), Yi et al. (2022), Zeng et al. (2011), Gao et al. (2010), Huang et al. (2009), Liu et al. (2017)
	5,2′-dimethoxy-6-(methoxymethyl)-7-hydroxy-isoflavonol	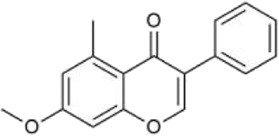	Anti-inflammatory effects and antioxidant activity Immunomodulatory regulation	Guan and Liu (2016), Wang et al. (2023)
Alkaloids	N-trans-feruloyltyramine, NTF	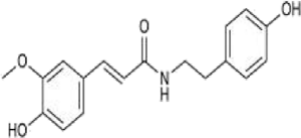	Antioxidant effects Suppression of pathological protein aggregation (notably Tau hyperphosphorylation and amyloid-beta [Aβ] fibrillization)	Gao et al. (2019), Othman et al. (2022), Tu et al. (2016)
	Indole-3-carboxaldehyde, I3A	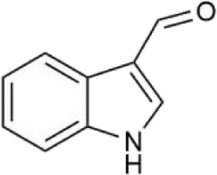	Suppression of pathological protein aggregation (notably Tau hyperphosphorylation and amyloid-beta [Aβ] fibrillization)	Purgatorio et al. (2020)
	betaine	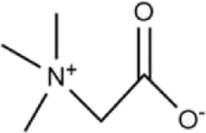	Regulation of neurotransmitter systems (particularly glycine-mediated inhibition and glutamine-glutamate cycling) Attenuation of apoptotic pathways Anti-inflammatory effects and antioxidant activity Inhibition of pathological protein aggregation (notably Tau hyperphosphorylation and amyloid-beta [Aβ] oligomerization)	Gao et al. (2019), Knight et al. (2017), Kunisawa et al. (2015), Purgatorio et al. (2020), Sun et al. (2017), Tu et al. (2016), Xie et al. (2016), Zhang and Jia (2023)

Although a substantial amount of experimental data indicates that *Achyranthes aspera* has certain therapeutic effects on neurodegenerative diseases, existing research still has some limitations. Currently, most studies on neurodegenerative diseases focus on individual active components. Therefore, future research could explore the synergistic effects among these components. Additionally, Although *Achyranthes aspera* has been used in traditional medicine for a long time, large-scale clinical studies are still needed to validate its pharmacological effects and mechanisms in humans. Furthermore, traditional Chinese medicines often exhibit weak indirect agonist activity, leading to insufficient bioavailability in the body. Future efforts should focus on discovering advanced drug delivery systems suitable for *Achyranthes aspera* to enhance the bioavailability of its active components, thereby more scientifically elucidating the correlation between drug actions and disease mechanisms.
